# Dysbiotic lung microbial communities of neonates from allergic mothers confer neonate responsiveness to suboptimal allergen

**DOI:** 10.3389/falgy.2023.1135412

**Published:** 2023-03-10

**Authors:** Jeffery C. Bloodworth, Aki Hoji, Garen Wolff, Rabindra K. Mandal, Nathan W. Schmidt, Jessy S. Deshane, Casey D. Morrow, Kirsten M. Kloepfer, Joan M. Cook-Mills

**Affiliations:** ^1^ Herman B Wells Center for Pediatric Research, Department of Pediatrics, Indiana University School of Medicine, Indianapolis, IN, United States; ^2^ Department of Microbiology and Immunology, Indiana University School of Medicine, Indianapolis, IN, United States; ^3^ Division of Pulmonary, Allergy and Sleep Medicine, Department of Pediatrics, Indiana University School of Medicine, Indianapolis, IN, United States; ^4^ Division of Pulmonary, Allergy and Critical Care Medicine, University of Alabama at Birmingham, Birmingham, AL, United States; ^5^ Department of Cell, Developmental and Integrative Biology, University of Alabama at Birmingham, Birmingham, AL, United States

**Keywords:** allergy, neonates, lung, microbiome, tocopherol

## Abstract

In humans and animals, offspring of allergic mothers have increased responsiveness to allergens. This is blocked in mice by maternal supplementation with α-tocopherol (αT). Also, adults and children with allergic asthma have airway microbiome dysbiosis with increased Proteobacteria and may have decreased Bacteroidota. It is not known whether αT alters neonate development of lung microbiome dysbiosis or whether neonate lung dysbiosis modifies development of allergy. To address this, the bronchoalveolar lavage was analyzed by 16S rRNA gene analysis (bacterial microbiome) from pups of allergic and non-allergic mothers with a basal diet or αT-supplemented diet. Before and after allergen challenge, pups of allergic mothers had dysbiosis in lung microbial composition with increased Proteobacteria and decreased Bacteroidota and this was blocked by αT supplementation. We determined whether intratracheal transfer of pup lung dysbiotic microbial communities modifies the development of allergy in recipient pups early in life. Interestingly, transfer of dysbiotic lung microbial communities from neonates of allergic mothers to neonates of non-allergic mothers was sufficient to confer responsiveness to allergen in the recipient pups. In contrast, neonates of allergic mothers were not protected from development of allergy by transfer of donor lung microbial communities from either neonates of non-allergic mothers or neonates of αT-supplemented allergic mothers. These data suggest that the dysbiotic lung microbiota is dominant and sufficient for enhanced neonate responsiveness to allergen. Importantly, infants within the INHANCE cohort with an anti-inflammatory profile of tocopherol isoforms had an altered microbiome composition compared to infants with a pro-inflammatory profile of tocopherol isoforms. These data may inform design of future studies for approaches in the prevention or intervention in asthma and allergic disease early in life.

## Introduction

Allergic asthma is the most common chronic airway disease in children. With the increase in prevalence of allergic diseases, approaches to limit development of allergy early in life are needed (1–3). A maternal history of allergic disease remains the greatest risk factor for development of allergies and asthma in offspring (4). Although mothers and their children occupy the same homes with the same pollutants and environmental contaminants and allergens, when controlling for these factors, there is increased sensitivity to development of allergy in the offspring of allergic mothers. Moreover, maternal transmission of reactivity to allergen in the offspring is not specific for the type of allergen in patients and animal models (5–11). Consistent with this non-specificity for type of allergen, transfer of splenic dendritic cells (DCs), but not macrophages, from neonatal mice of allergic mothers transfers allergen responsiveness to recipient neonates from non-allergic mothers (4, 12). Neonates from allergic mothers have increased lung DC subsets, including monocyte-derived DCs (mDCs) and resident DCs (rDCs) but no change in regulatory DCs (pDCs or CD103 + DCs) (13, 14). The development of responsiveness to allergen results from complex interactions with environmental factors, including allergens and dietary lipids (13, 14).

We have demonstrated that responsiveness to allergen by neonates of allergic mothers is modifiable by the dietary lipids α-tocopherol (αT) and γT in maternal diets (13, 14). In adult mice, we demonstrated that γ-T elevates allergic responses to chicken egg ovalbumin (OVA) (15, 16) and to house dust mite extract (HDM) (17) and that γT potently ablates the anti-inflammatory benefit of α-T during allergic responses (15, 16). We also demonstrated that dietary supplementation of allergic mothers during pregnancy and nursing with αT inhibited, whereas γT increased development of allergen (OVA) responsiveness and DC numbers in the offspring of allergic mothers (13, 14). During allergic inflammation, tocopherols function as anti-oxidants and regulate signal transduction during DC differentiation and leukocyte transendothelial migration (18–20). We demonstrated that tocopherols regulate cell signal transduction by binding to a regulatory domain of protein kinase C; when bound to PKC, αT is an antagonist and γT is an agonist of PKCα (15, 18, 20–23).

In humans, we demonstrated that plasma with low αT & >10 µM γT associates with lower lung function in children (24) and in adults (25). Based on the prevalence of serum γ-T > 10 µM in adults in the USA and adults in the 2011 USA census, up to 4.5 million U.S. adults had >10 µM serum γ-T and may have had 500 ml lower FEV1 and FVC (25). We also demonstrated that γ-T associated with increased odds for asthma in China (17). It is reported that patients with asthma or allergy have low levels of α-tocopherol (26–29), suggesting that an increase in α-tocopherol may be necessary, in combination with other regimens, to decrease allergic disease. We also reported that higher plasma αT and lower γT concentrations in children at 3 years of age associate with better lung function at 6–19 years of age in project VIVA (24). Together the preclinical studies in mice and humans indicate that a higher plasma γT concentration is a pro-inflammatory tocopherol profile and a higher plasma αT with lower γT concentration is an anti-inflammatory tocopherol profile with regards to allergic inflammation and lung function. Thus, the preclinical studies and data for clinical associations suggest that tocopherols may modify mediators that regulate the development of allergic disease.

It is suggested that risk for allergic disease in humans is associated with *in utero* and early exposures to environmental factors (30). Microbiota are acquired from the environment *in utero* and as neonates. Since the advent of non-culture-dependent microbe characterization, the lungs have been known to harbor commensal and pathogenic microbes (31). The airways are colonized by a diverse range of bacterial, archaeal, protozoal and fungal microorganisms known collectively as the airway microbiome. Microbes occupy the airways during health and disease, but the abundance and diversity of microbes is altered during lung diseases including asthma. Briefly, microbes of the Proteobacterium phylum are elevated during allergic asthma, including members of the *Streptococcus*, *Haemophilus*, and *Moraxella* genera (32–35). Streptococcus colonization during early life is a strong predictor of allergic asthma development (35). Alterations in the microbiota have a profound association with allergic asthma development during childhood (35). It is not known whether αT supplementation modifies the lung microbiome in offspring of allergic mothers. Moreover, it is not known whether lung microbiome of offspring of allergic mothers affects the development of responsiveness to allergen in the offspring lung.

We report that in mice, the lung microbiome of offspring of allergic mothers is altered before allergen challenge of the neonate. This is blocked by maternal supplementation with αT. Moreover, transfer of the lung microbial communities of offspring of allergic mothers to offspring of non-allergic mothers confer responsiveness to allergen. In infants, a pro-inflammatory profile of tocopherol isoforms associates with an altered airway microbiome.

## Methods

### Animals

Adult C57BL/6 female and male mice were from Jackson Laboratory, Bar Harbor, Maine and maintained under specific pathogen free (SPF) conditions at Indiana University Lab Animal Resource Center. C57BL/6 mice are used in this study because C57BL/6 mice have been vital for our studies of mediators that regulate the development of allergic responses by offspring of allergic mothers (13, 14, 36). The studies were approved by the Indiana University Institutional Review Committee for animals.

### Tocopherol and basal diets

αT is necessary for mouse and human placental development (37, 38). Standard basal mouse chow diet contains 45 mg αT/kg of diet and 45 mg γT/kg of diet and supports fetal development in mice. Supplemented αT diets contain 250 mg αT/kg of diet and 45 mg γT/kg of diet (13, 14). Translation of mouse basal α-T doses to humans is calculated as we previously described (page 173 of 39). Taking into account differences in food consumption and metabolism (39), a 45 mg αT/kg of diet for mice is 57 mg αT/day for human adults. For healthy adult humans, 15 mg αT/day is recommended, but asthmatics have low plasma αT (26–29). The 250 mg αT/kg of diet for mice is 285 mg αT/day for human adults, which is well below upper safety limits of 1,000 mg αT/day in human pregnancy and near the 268 mg (400IU) d-αT in pre-eclampsia pregnancy trials (40–45). A relevant dose is a dose that achieves similar fold changes in tissues in mice and humans.

For supplementation of diets with tocopherol, D-α-tocopherol (>98% pure) from Sigma was sent to Dyets, Inc (Bethlehem, PA) to produce the diets with 250 mg αT/kg of diet (catalog#103373) (13, 46). The purity of these tocopherols that were used to make the diets and the tocopherol concentrations in the diets were confirmed by HPLC with electrochemical detection as previously described (13, 46). These αT supplemented diets increase tocopherols 3-fold in mothers and pups (13, 14, 47–49). This is similar to fold tissue changes achievable in humans (15, 16, 21–23, 46).

### Allergens

**Figure 1:**
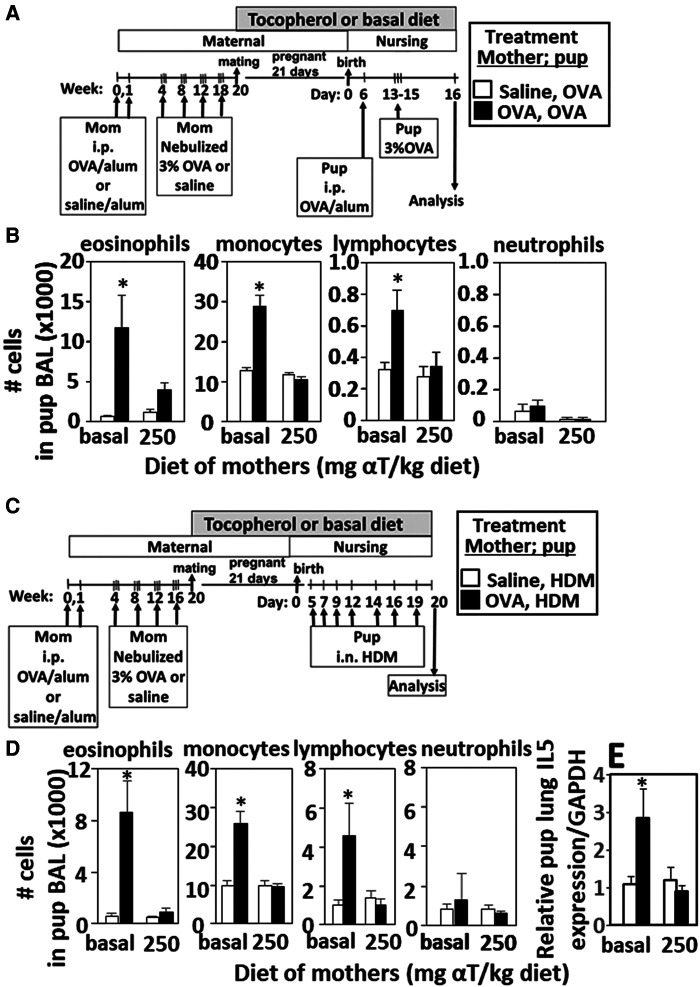
Enhanced responsiveness to challenge with HDM or OVA by pups of mothers with allergy was inhibited by maternal supplementation with α-tocopherol. The allergen of the mother and offspring can differ. (**A,C**) Allergic and non-allergic mothers received basal diet or diet supplemented with αT (250 mg αT/kg of diet) during pregnancy and nursing. Timeline for allergen-sensitization and allergen-challenge of mothers and offspring. (**B,D**) Pup BAL eosinophils, monocytes, lymphocytes and neutrophils. (**E**) Relative IL-5 mRNA expression in lungs of HDM-challenged pups of allergic and non-allergic mothers with basal or αT-supplemented diets. BAL, bronchoalveolar lavage. *n* = 8–10 mice/group. Saline treated pups did not have allergic inflammation (data not shown). *p < 0.05.

In our studies, pups received the same allergen or different allergen than the mother because in mice (5–11) and humans (4), allergen responses by offspring are not specific to the allergen to which the mother had been exposed. We induced allergic lung inflammation in mothers or pups with OVA or HDM. OVA is a well-characterized model purified allergen that, in humans, can also be inhaled when exposed to powered egg or when gasping during egg allergic reactions. HDM extract is a model environmental allergen from Greer and has been used in allergy shot induction of tolerance in humans. Mothers with allergic responses and allergic inflammation at the time of mating (15, 46, 50) are mated to non-allergic fathers (13, 14). On gestational day 18 (GD18) [during time of fetal hematopoiesis], we collect mother plasma, placentas, and fetal livers (site of hematopoiesis in the fetus) (13, 14). To assess offspring development of allergy, pups receive a suboptimal allergen sensitization/challenge protocol (Figure 1) (13, 14). There are no differences by sex so data include both sexes (13, 14).

### Separation of microbiota from mouse BAL

For lung microbiota transfers and microbiota taxa analyses, the microbes were *separated* from the BAL by differential centrifugation as follows: the BAL was centrifuged for 10 min at 1200 rpm to pellet host cells and supernatant collected. This supernatant was centrifuged at 10 min at 15,000 rpm (500×g) to pellet the microbiome. The supernatant, which contains soluble proteins and mucins (51), was removed and pellet contains the microbiota. The microbiota were 96 ± 0.7% viable as determined by the Biotium Bacterial Viability and Gram Stain Kit with analyses by flow cytometry. Also for visualization of microbiota, separated BAL microbial communities from allergen-challenged pups of allergic and from non-allergic mothers were suspended in a minimal volume, fixed in a small spot on a glass slide, stained with a gram stain for bacteria, and analyzed by microscopy.

### OVA administration, lung microbiota transfers and analysis of inflammation

C57BL/6 female mice were maintained on chow diet. The mice were sensitized by intraperitoneal injection (200 μl) of OVA grade V (Sigma-Aldrich Co.) (5 μg)/alum (1 mg) or saline/alum (1 mg) on days 0 and 7 (13, 14). The mice were exposed to nebulized saline or 3% (w/v) OVA in saline for 15 min on 3 consecutive days at 8, 12, and 16 weeks of age and then mated. The pregnant and nursing dams received basal diet (45 mg αT/kg of chow) or αT-supplemented diet (250 mg αT/kg of chow) as indicated in the figures.

In experiments with transfer of lung microbial communities, BAL was collected from neonates at PND5. The BAL microbial communities were separated by differential centrifugation For transfer of microbial communities, the pelleted donor microbiota were suspended in saline and immediately administered to lungs of recipient pups to most closely represent the donor microbiome levels and abundance. The few hours between collection of BAL microbiota from donor pups to administration to recipient pups was within the timeframe described for survival of aerobic microbes, as these aerobic microbes can survive for days in PBS (52). The BAL microbial communities of 2 donors were combined for each recipient and administered in 10 µl intranasally to each PND4 recipient pup for adequate inoculation, similarly to studies by others with viral inoculation of neonates (53).

For allergen challenge of the pups, six-day old pups were **sub-optimally** sensitized by treating with only one 50 µl i.p. injection (rather than two injections) of 5 µg OVA/1 mg alum (13, 14). At 13, 14, and 15 days old, the pups were challenged for 15 min with 3% OVA. At 16 days old, the pups were weighed, euthanized and tissues collected. Pup bronchoalveolar lavage (BAL) cells were stained and counted as previously described (13, 14). OVA-specific IgE was determined by ELISA as previously described (13, 14).

### 16S rRNA gene analysis of mouse BAL

For microbiome analyses, the microbes were *separated* from the BAL by differential centrifugation. To limit confounding contributions from contaminant bacteria during collection and sequencing reagents (54, 55), data from the BAL microbiome of pups of allergic mothers were compared to control BAL groups and *N* = 8–10 pups from 3 to 4 mothers per group. The same sterile reagents were used within an experiment. 16S rRNA gene amplicons were generated *via* PCR amplification using primers 5′TATGGTAATTGTGTGCCAGCMGCCGCGGTAA3′ and 5′AGTCAGTCAGCCGGACTACHVGGGTWGCTAAT3′ (56). The full sequencing protocol is published by Kumar et al. (56) The 16S rRNA gene sequencing was performed on the Illumina MiSeq platform. The ASV table for the mouse microbiome studies was generated by an analysis pipeline using CLC Genomic Workbench Microbial Module (CLCGW-MM). This includes the preprocessing of V4 16S amplicon (250 bp) reads, mapping to SILVA 16S v.132 SSURef, and filtering of initial ASVs (relative abundance > 1 × 10^−^5) as described in the University of Alabama at Birmingham protocol (56, 57). The nomenclature for Bacteroidetes has recently been updated to Bacteroidota (58), therefore we are using these synonymously in this manuscript. Differential abundant analysis was done by a built-in function in the CLCGW-MM, which generated FDR and log2fold differences in the taxa between two comparison groups. Data are shown as % abundance of 16S rRNA gene amplicon counts of total counts within specific taxonomic levels. Alpha and beta diversity analyses were performed using Quantitative Insights into Microbial Ecology v2 2022.8 (59, 60). Principal component analysis was performed using EMPeror (61).

### qPCR analysis of cytokines and chemokines

Total RNA was isolated from 50 to 100 mg lung tissue using the QIAGEN RNeasy Mini Kit (catalog #74136). cDNA was prepared using a MMLV Reverse Transcriptase kit (QuantaBio, catalog #95047) and analyzed by PCR on an ABI 7300 Thermal Cycler (Applied Biosystems). Taqman probes and Taqman Universal Master Mix were used as directed (Applied Biosystems, catalog #4304437). Taqmanprobes used were GAPDH (catalog #4331182) and MUC5AC (catalog#4331182). IL-5, IL-13, IL-33, and CCL11 expression levels were quantified using SsoAdvanced Universal SYBR Green (Biorad catalog# 1725271) with the following primers obtained from Integrated DNA Technologies.

**Table:** 

Target	Forward primer sequence	Reverse primer sequence
CCL11	TGTAGCTCTTCAGTAGTGTGTTG	CTTCTATTCCTGCTGCTCACG
GAPDH	GTGGAGTCATACTGGAACATGTAG	AATGGTGAAGGTCGGTGTG
IL-33	AATCACGGCAGAATCATCGAGAAA	GGAGCCAGAGGATCTCCGATT
IL-13	CCAGGGCTACACAGAACCCG	GCTCTTGCTTGCCTTGGTGG
IL-5	ACTGTCCGTGGGGGTACTGT	CCTCGCACACTTCTCTTTTTGG

### INHANCE cohort

INHANCE cohort (62, 63) is an urban cohort (birth to 18 months of age) in the Indianapolis area (*n* = 180, 70% Black or mixed-race Black; NIH K23 AI135094-01 PI Kloepfer). Of these 180 infants, 43 of the 3–5 months of age infants and 50 of the 12–18 months of age infants had nasal 16S microbiome data (62, 63) and sufficient plasma volume available for tocopherol analyses by HPLC.Human 16S rRNA gene sequencing data were analyzed using Quantitative Insights into Microbial Ecology v2 2022.8 (59, 60). Human sequences were aligned with the SILVA 138.1 taxonomy database (57). Also serum tocopherol concentrations at 3–5 months or 12–18 months of age were measured by HPLC with electrochemical detection (64) as previously described (24). Because we have demonstrated that in children and adults that better lung function associates with increasing αT when gamma-tocopherol (γT) concentrations are lower (24, 25, 65–67), the INHANCE cohort infants were placed in groups based on below or above median αT and median γT concentrations (64 and manuscript in preparation) withQ1 (high γT, low αT), Q2 (high γT, high αT), Q3 (low γT, low αT) and Q4 (low γT, high αT). Q4 has an anti-inflammatory profile of tocopherols and Q2 has a pro-inflammatory profile for tocopherol isoforms for allergic lung inflammation and lung function as in our previous reports in children and adults (13, 14, 24, 25, 66, 68).

#### Data availability

**Figure 2:**
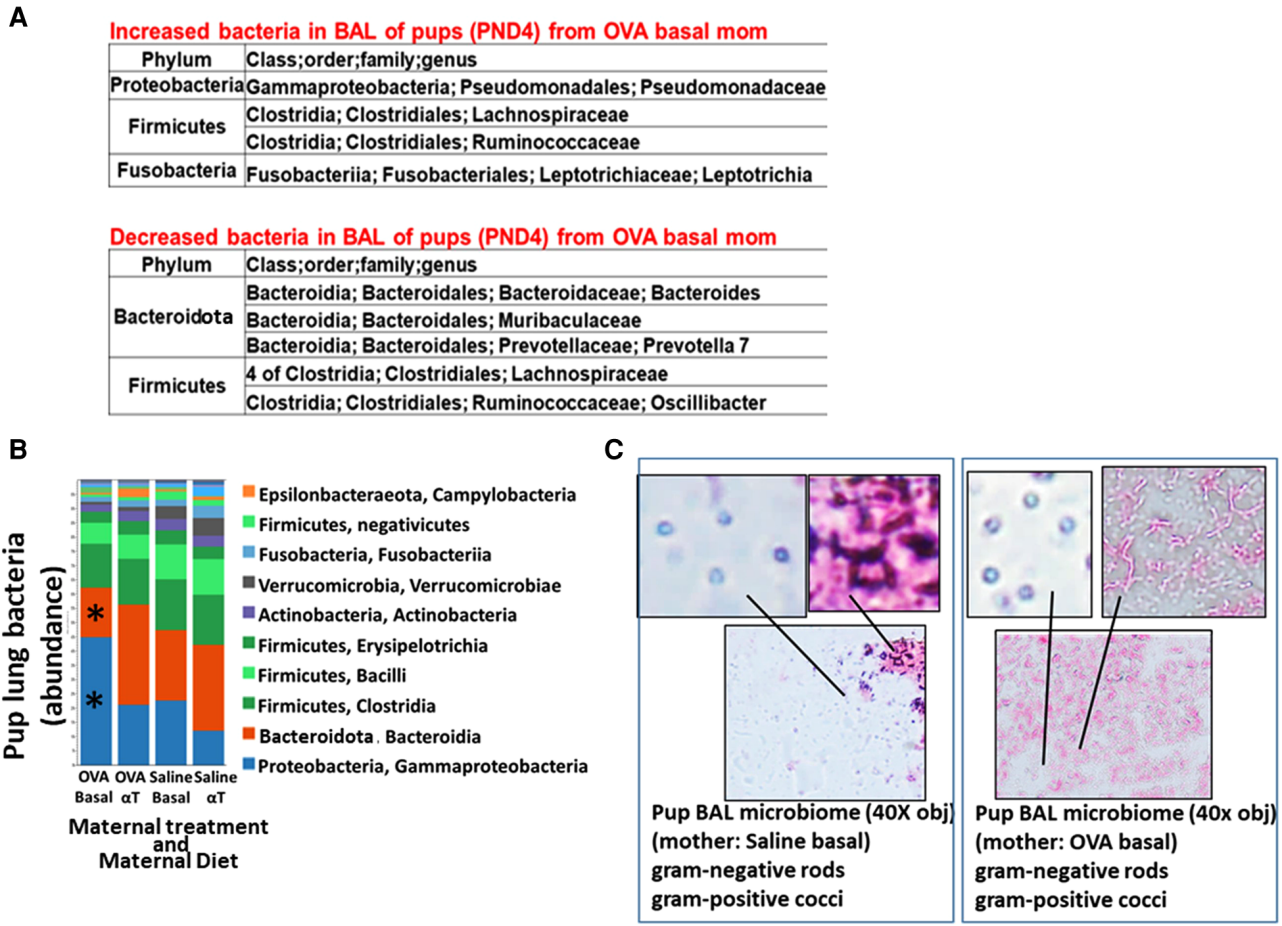
Pups of allergic mothers have altered lung bacteria microbial composition. Mouse treatments were as in (Figure 1A). BAL microbiota from pups at (**A**) PND4 and (**B,C**) 24 h after OVA-challenge (PND16) was separated and analyzed by 16S rRNA gene sequencing and a microbiome analysis pipeline. (**A**) At PND4, before allergen exposure, there was increased Proteobacteria and decreased Bacteroidota in the lungs of offspring of allergic mothers (log2FC > 0.6, FDR < 0.1) as compared to offspring of non-allergic mothers with basal diet. (**B**) ASV table of the relative abundance of phyla within the total pup BAL bacterial microbiome. *p < 0.05 compared to other groups. (**C**) BAL microbiota from pup BAL PND16 were separated and concentrated by differential centrifugation as in the methods, suspended in minimal volume for fixation in a small spot on a glass slide and stained with gram stain from bacteria. Representative images of lung microbiota are shown.

**Figure 3:**
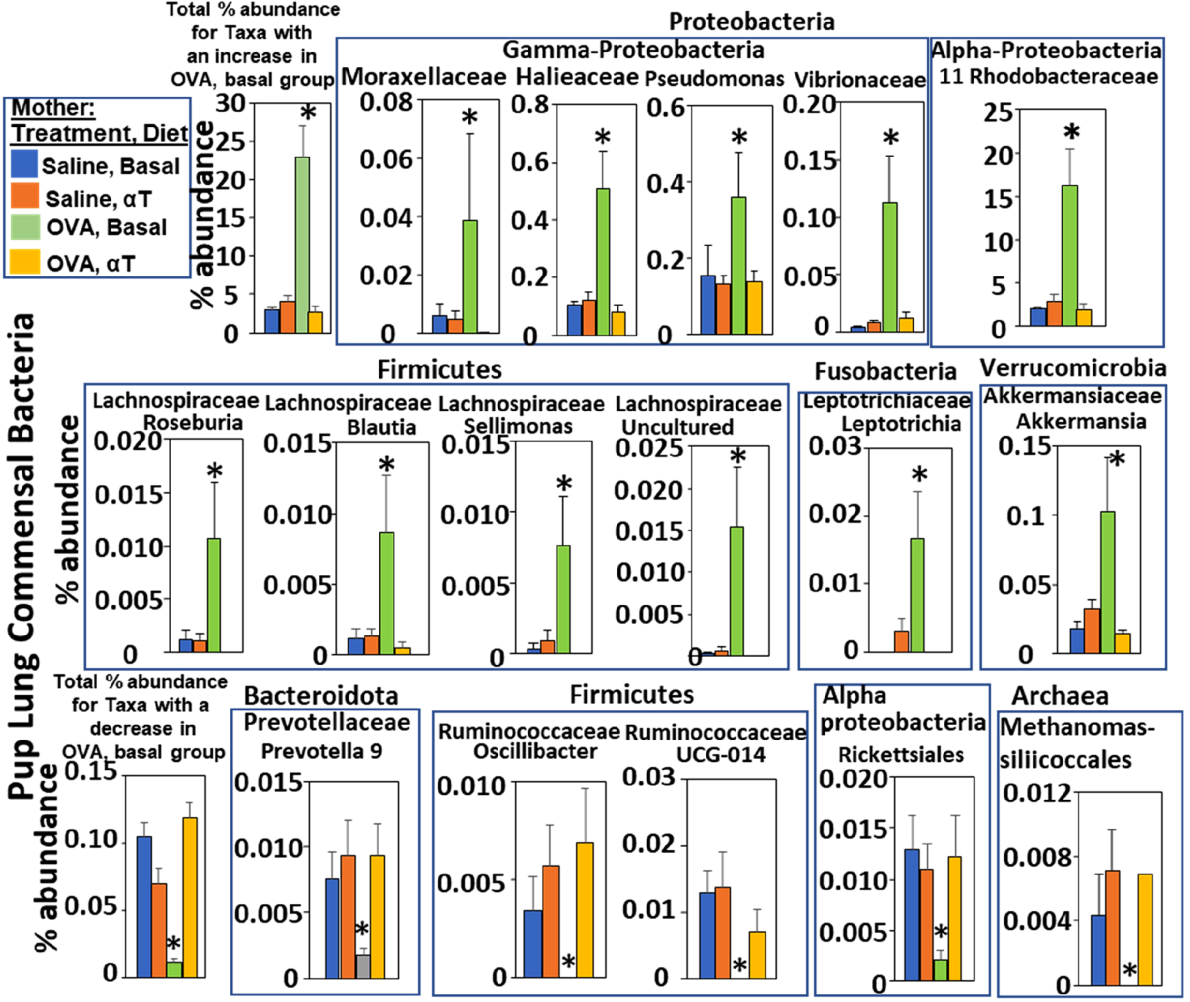
Pups of allergic mothers have altered bacteria microbiome. Mouse treatments were as in (Figure 1A). Pup BAL microbiota were separated and analyzed by 16S rRNA gene sequencing at PND16. Shown are the % abundance for pup BAL bacteria with a significant difference in the OVA/Basal group compared to the other groups. *p < 0.05.

**Figure 4:**
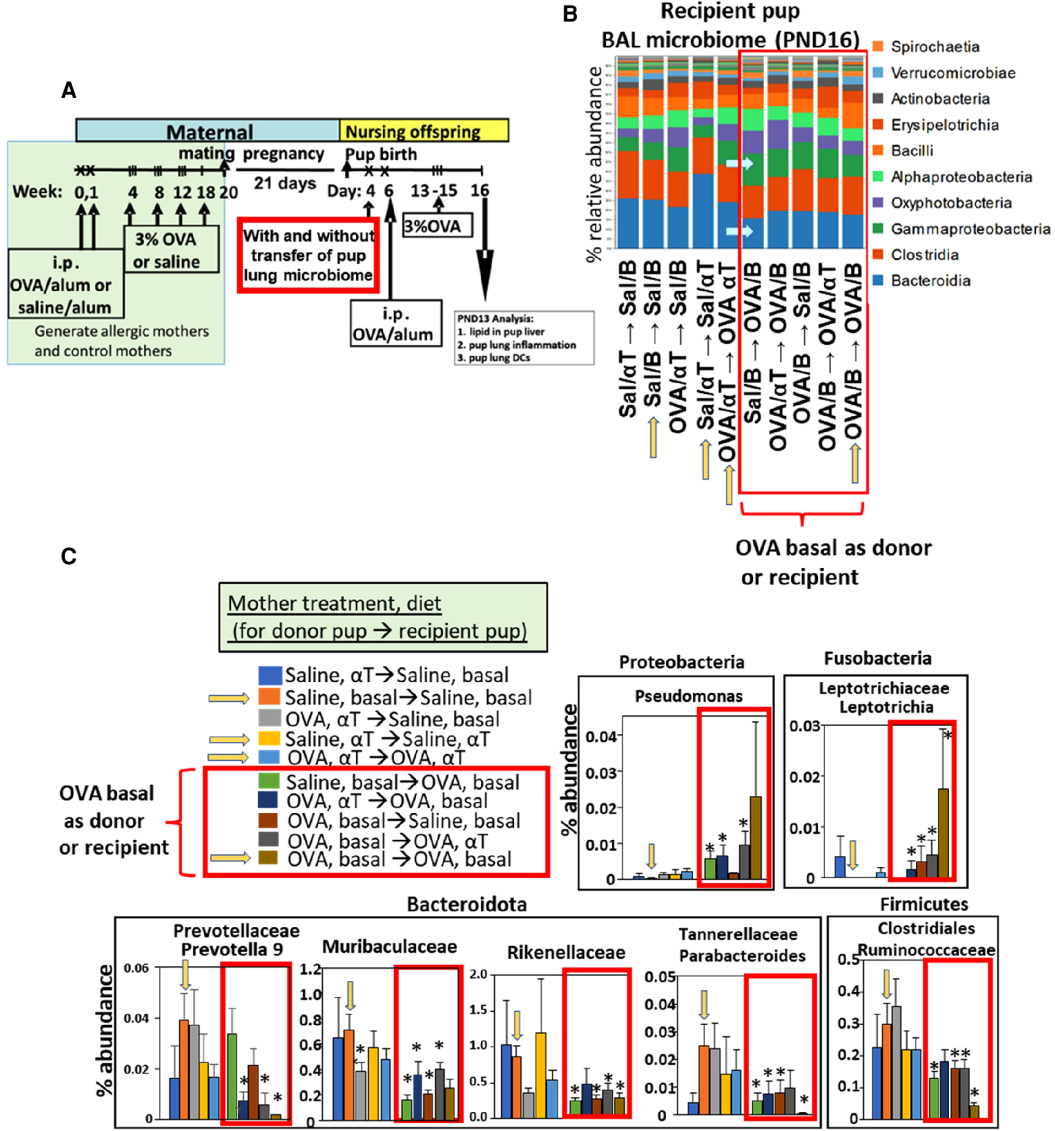
After intranasal microbiome transfers and airway allergen challenge, there was pup BAL microbiota with increased Proteobacteria and decreased Bacteroidota taxa for pups that were either recipient pups of mothers in the OVA,basal group or were pups receiving microbiome from pups of mothers in the OVA,basal group. (**A**) Timeline for treatment of mothers and pups. (**B**) Donor BAL microbiome was administered intranasally in 10 µl to PND4 recipient pups (as indicated in figures as the group of pups providing donor BAL microbiome for transfer into a recipient group of pups, i.e., donor → recipient group). **Yellow arrows on the x-axis** are those groups with donor and recipients within the same group. In **RED BOX** are groups with recipient or donor microbiota of PND16 pups of allergic mothers (OVA/basal). Blue arrows within panel B indicate that Bacteroidota are decreased and Gamma-Proteobacteria are increased in groups in red box. *N* = 8/group. In panels B,C only, the OVA was in 0.09% saline; nevertheless, it did not alter the fold effect on BAL cell inflammation which is included in (Figure 5) with data from 7 microbiome transfer experiments. (**C**) In **RED BOX** are recipient or donor microbiota of PND16 pups from allergic mothers (OVA/basal). Data are presented as percent abundance of bacteria taxa. *, *p* < 0.05 as compared to Saline,basal → Saline,basal group (yellow arrow in graphs in C). Sal/B, saline-treated mother with basal diet. Sal/αT, saline-treated mother with αT-supplemented diet. OVA/B, OVA allergen-treated mother with a basal diet. OVA/αT, OVA allergen-treated mother with αT-supplemented diet.

**Figure 5:**
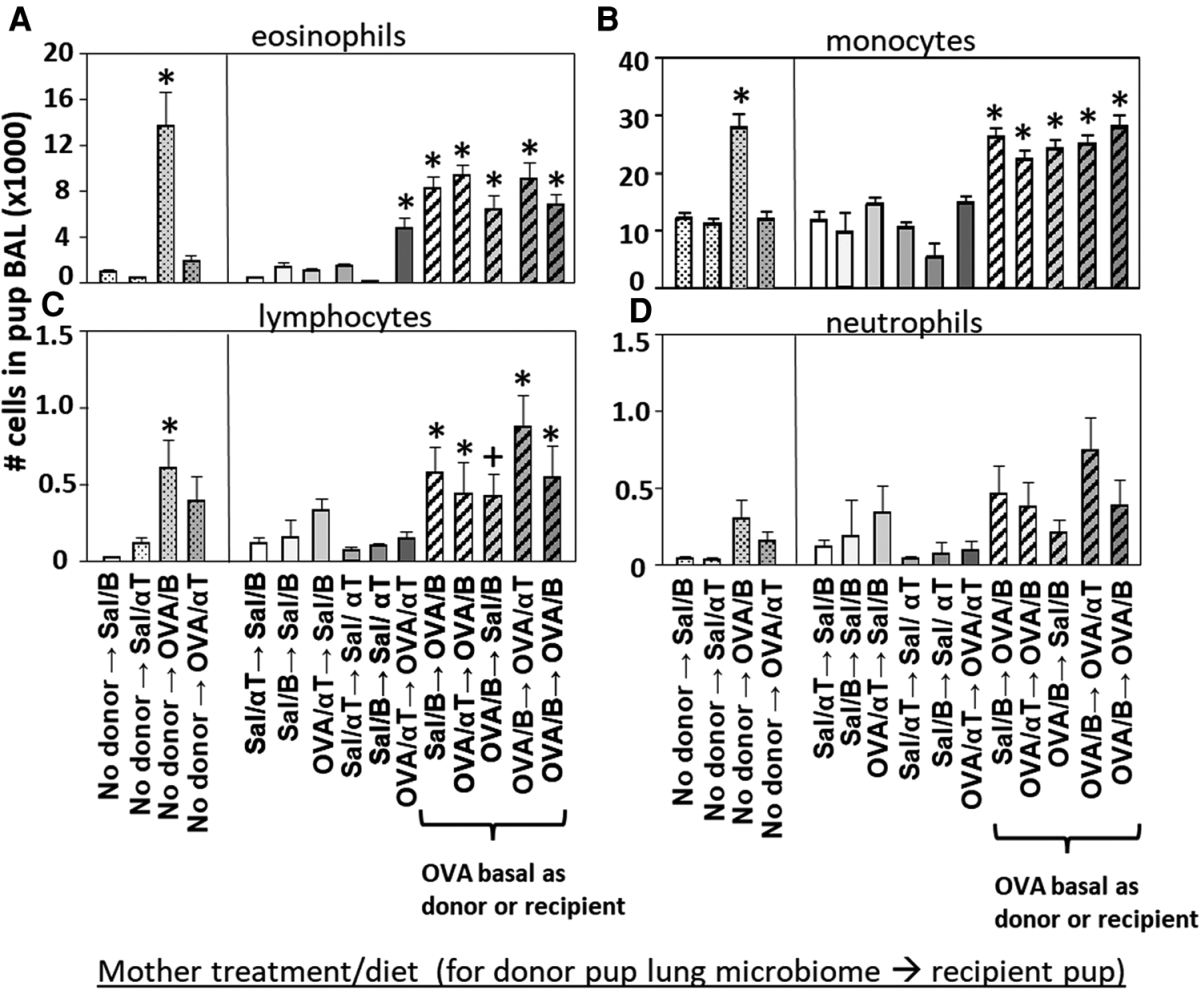
Recipient or donor microbiota from pups of allergic mothers (OVA,basal) conferred responsiveness to allergen in the recipient pups (red box). Mice were treated as in timeline in (Figure 4A) BAL (**A**) eosinophils, (**B**) monocytes, (**C**) lymphocytes, and (**D**) neutrophils are presented as mean ± SEM. Data are from 7 experiments. *N* = 10–36/group. Sal/B, saline-treated mother with basal diet. Sal/αT, saline-treated mother with αT-supplemented diet. OVA/B, OVA allergen-treated mother with a basal diet. OVA/αT, OVA allergen-treated mother with αT-supplemented diet. *p < 0.05 as compared to the saline,basal → saline,basal group., +*p* < 0.1 as compared to no donor → Saline/basal group.

The raw fastq files of the 16S rRNA analysis from mouse BAL in (Figures 2–5) are deposited as NCBI BioProject repository, accession number ID PRJNA925891. The raw fastq files of the 16S rRNA analysis in INHANCE cohort in (Figure 8) are deposited as NCBI BioProject repository, accession number ID PRJNA928382.

### Statistics

Data in the figures were analyzed by a one-way ANOVA followed by Tukey's or Dunnett's multiple comparisons test (JMP software, SAS Institute). Data in figures are presented as the means ± the standard errors. Data include both genders because there were no differences in outcomes by gender (data not shown). For analyses of the 16S microbiome from INHANCE cohort infants 3–5 months and 12–18 months of age, cutoffs were set for the data, including removal of 3 samples with insufficient ASV detection (<4% total ASV reads/sample compared to other samples), removal of ASV's that had less than 6/43 samples with reads, and to address extension of findings in mice to human, phyla were included in analyses for phyla observed in the mouse models. These participants were placed in 4 groups based on the median αT and γT concentrations (64). Then an abundance cutoff was set at >0.003% abundance for the sum of the ASV averages for the groups. This yielded 181 ASV for the 3–5 months age and 217 ASV for the 12–18 months age. Based on predetermined results in mice with αT supplementation and low γT, analyses were made in comparison to the group Q4 which had a serum αT concentration above the median and a serum γT below the median concentration. There was no formal adjustment for multiple testing because the analyses were selected based on preclinical mechanistic microbiome outcomes. Furthermore, the associations tested were established *a priori* at the onset of the project with microbiome as the primary analysis with tocopherol isoforms.

## Results

### Enhanced responsiveness to challenge with HDM or OVA by pups of mothers allergic to OVA is inhibited by dietary supplementation of the mother with α-tocopherol

Pups of allergic mothers respond to suboptimal OVA sensitization with allergen and this allergen responsiveness of the offspring is reduced by dietary supplementation of the mother with αT during pregnancy and nursing (13, 14). It is not known whether the pups of allergic mothers also respond to HDM and whether this is modified by αT. Pups of OVA-challenged mothers were responsive to suboptimal sensitization and challenge with OVA (Figures 1A,B) or HDM (Figures 1C,D) with increased numbers of leukocytes (Figures 1B,D) and this was blocked by maternal dietary supplementation with α-tocopherol as compared to pups from allergic mothers with a basal diet (Figures 1B,D). OVA increased numbers of eosinophils, monocytes and lymphocytes in the BAL (Figures 1B,D), OVA-specific IgE (13, 14), and lung cytokines (13, 14) in pups of allergic mothers as compared to pups of non-allergic mothers. HDM challenge of pups of allergic mothers (Figure 1C) also increased numbers of eosinophils, monocytes and lymphocytes in the BAL (Figure 1D) and lung IL-5 expression (Figure 1E). It has been demonstrated that pups of allergic mothers that are challenged with saline do not have allergic lung inflammation and that BAL cell numbers are similar to allergen-challenged pups of non-allergic mothers.(5) There were no sex differences in pup weight or eosinophilia as we previously reported (13, 14), so data include both sexes.

### Pups of allergic mothers but not pups of non-allergic mothers exhibited lung bacterial microbiome dysbiosis

The airway microbiome is altered in adult humans with allergic asthma and in adult mice with allergic lung inflammation (69). This airway microbiome dysbiosis in adults has an increased abundance of Proteobacteria and decreased Bacteroidota (69–71). It is not known whether the lung microbiome is altered in pups of allergic mothers. It is also not known whether the lung microbiome plays a role in regulation of airway response to allergen. Interestingly, at PND4 before pup exposure to allergen, the BAL of pups of allergic mothers with a basal diet had increased abundance of Proteobacteria and decreased abundance of Bacteroidota (log2FC > 0.6, FDR < 0.1) as compared to pups of non-allergic mothers with a basal diet (Figure 2A). To assess whether allergen alters the bacterial microbiome of pups, the pups of allergic mothers and pups of non-allergic mothers were challenged with a purified allergen. OVA was used as a purified protein allergen, thereby avoiding contaminant bacterial 16S in the extracts from HDM. The microbiota, that was separated from the BAL of allergen-challenged PND16 pups, contained gram negative and gram positive microbiota as determined by gram-staining of BAL bacteria fixed to glass slides (Figure 2C). The 16S analyses of the PND16 BAL microbiota of allergen-challenged pups of allergic mothers demonstrated an increase in abundance of Bacteriodota and decrease in abundance of Proteobacteria (Figure 2B). There were increases in several taxa within the Proteobacteria, Firmicutes, Fusobacteria and Verrucomicrobia, but decreases in taxa with the Bacteroidota and several other Firmicutes, Proteobacteria and Archaea as compared to pups of non-allergic mothers and as compared to pups of allergic mothers supplemented with αT (Figure 3).

### Transfer of the BAL microbial community of pups of allergic mothers to pups of non-allergic mothers sustained the donor microbiome in the recipient pups

Neonate bacterial load increases over PND0-14 (72). To address a potential function for the microbiome dysbiosis in allergen responsiveness, the microbial community was obtained from the BAL of PND4 pups without allergen exposure. The donor PND4 BAL microbial community was separated from the BAL and transferred intranasally to recipient PND4 pups. Then, the PND4 pups without donor microbiota and the PND4 pups that received the microbiota transfers were challenged with allergen (Figure 4A). For the transfers, the pup groups are designated as maternal treatment of the donor pups → maternal treatment of the recipient pups. The donor sample 16S microbiome had increased Proteobacteria and decreased Bacteroidota taxa in the lungs of offspring of allergic mothers (log2FC > 0.6, FDR < 0.1) (Figure 2A).

The PND4 donor microbiota were also analyzed for alpha-diversity and beta-diversity. The PND4 donor microbiota groups had a similar Shannon within-group alpha-diversity index; the Shannon Index incorporates total number of bacterial species and relative differences in the abundance of various species in the microbiota community of a group (Supplementary Figure S1A). For beta-diversity analysis, the donor groups did not separate in the Principal Component Analysis (PCA) of the Unweighted Unifrac and Weighted Unifrac between-group beta-diversity analysis of bacterial microbiota (Supplementary Figures S2B,C, left panels); the weighted-Unifrac analysis incorporates only the relative abundance of taxa shared between samples and the unweighted-Unifrac analysis incorporates only the presence/absence of taxa between groups. In contrast, when incorporating both overall abundance per sample and abundance of each taxa of the microbiota communities by the Bray-Curtis beta-diversity distance analyses, there was clustering by PCA for the donor saline groups and for the donor OVA groups, which was unaffected by αT (Supplementary Figure S2A, left panel).

Alpha-diversity was also assessed for the PND16 pup microbiota from the BAL of allergen-challenged pups with and without microbiota transfer. Without the microbiota transfers, the BAL of allergen-challenged PND16 pups from allergic mothers (the no donor → OVA/B group in Supplementary Figure S1B) had decreased alpha-diversity as compared to the saline groups (the no donor → Sal/B group and the no donor → Sal/αT group) (Supplementary Figure S1B). With the microbiota transfers, the BAL of allergen-challenged PND16 pups of allergic mothers (OVA) as either donor or recipients (designated as microbiota donor → recipient pairs of pups) had reduced alpha-diversity (Supplementary Figure S1B, red box) as compared to several control groups, including the no donor → Sal/B, the Sal/B → Sal/B, the no donor → Sal/αT or the OVA/αT → OVA/αT (Supplementary Figure S1B).

Beta-diversity was assessed for the PND16 pup microbiota from the BAL of allergen-challenged pups with and without microbiota transfer. There was minimal separation of the PND16 groups in the PCA plot of the Unweighted Unifrac and Weighted Unifrac between-group beta-diversity bacterial microbiota analyses (Supplementary Figures S2B,C, right panels). In the PCA plot of the Bray-Curtis beta-diversity distance analyses of the allergen-challenged pups without donor microbiota transfers (Supplementary Figure S2A, right panel with cone-shaped symbols), there was some separation of the no donor → OVA/B group as compared to the other no donor groups. In the PCA plot of the Bray-Curtis beta-diversity distance analyses of the allergen-challenged pups that received donor microbiota transfers, there was unique clustering of microbiota from pups of allergic mothers with basal diet (OVA/B) as either donors or recipients (Supplementary Figure S2A, right panel with sphere-shaped symbols); these are the groups with allergic inflammation in Figure 5.

Notably, when either the BAL microbial community of the donor pup or the recipient pup was from an allergic mother with basal diet (OVA/B), the recipient pup BAL had an increase in abundance of the class Gamma-proteobacteria and decrease in abundance of the class Bacteroidia (Figure 4B), as compared to the saline/B → saline/B group of pups (Figure 4B). In Figure 4C, when the BAL microbial community was from a group with a donor pup or the recipient pup from an allergic mother with basal diet (OVA/B), there was an increase in a Proteobacteria and a Fusobacteria and a decrease in several Bacteriodota taxa and a Firmicute. These data suggest that the BAL microbial community of the pups of allergic mothers with basal diet was dominant.

### Transfer of the dysbiotic BAL microbial community of pups of allergic mothers to pups of non-allergic mothers conferred enhanced responsiveness to allergen in the recipient pups, demonstrating a functional role for the lung microbiome

The BAL cells were assessed for the pups in (Figure 4). Without microbiota transfers, the pups of allergic mothers had increases in BAL eosinophils, monocytes and lymphocytes (Figure 5), no-donor groups). After intranasal microbiome transfers and airway allergen challenge, there were increased numbers of BAL eosinophils, monocytes and lymphocytes in the pups that were either recipient pups of mothers in the OVA,basal group or were pups receiving microbiome from pups of mothers in the OVA,basal group (i.e. OVA/B as donor or recipient) as compared with the pups of the control saline/B → saline/B group (Figure 5). The donor → recipient pup groups without an OVA/B group in the donor or the recipients did not develop lung eosinophilia after allergen exposure (Figure 5). Interestingly, the mice were in a specific-pathogen-free facility and dysbiosis of the transferred microbial communities was sustained in recipient pups (Figure 4) and only exhibited in pups with allergic lung responses (Figure 5). These novel transfer studies demonstrate that the dysbiotic microbiome of pups of allergic mothers enhances pup responsiveness to allergen.

### The transferred BAL microbial community influenced induction of allergen-specific IgE and the allergen-induced expression of cytokines

**Figure 6:**
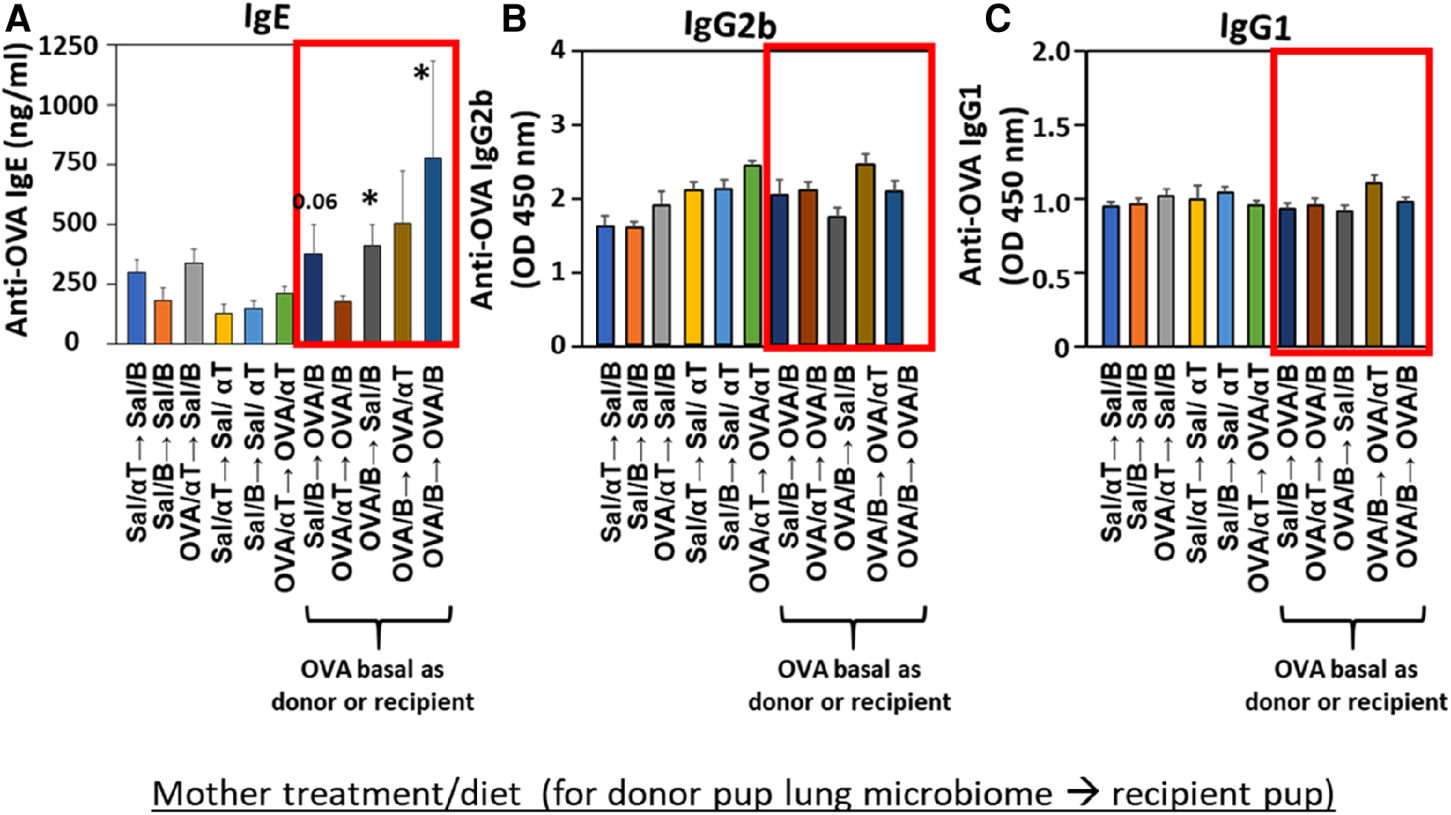
Recipient or donor microbiota from pups of allergic mothers (OVA,basal) conferred allergen sensitization with increased IgE but not increased IgG2b or IgG1 (red box). Mice were treated as in timeline in (Figure 4A). Serum (**A**) anti-OVA IgE, (**B**) anti-OVA IgG2b, and (**C**) anti-OVA IgG1 as determined by ELISA. Data are presented as mean ± SEM. *N* = 6–9/group. Sal/B, saline-treated mother with basal diet. Sal/αT, saline-treated mother with αT-supplemented diet. OVA/B, OVA allergen-treated mother with a basal diet. OVA/αT, OVA allergen-treated mother with αT-supplemented diet. *p < 0.05 as compared to the saline,basal → saline,basal group. +*p* < 0.1 as compared to no donor → Saline/basal group.

**Figure 7:**
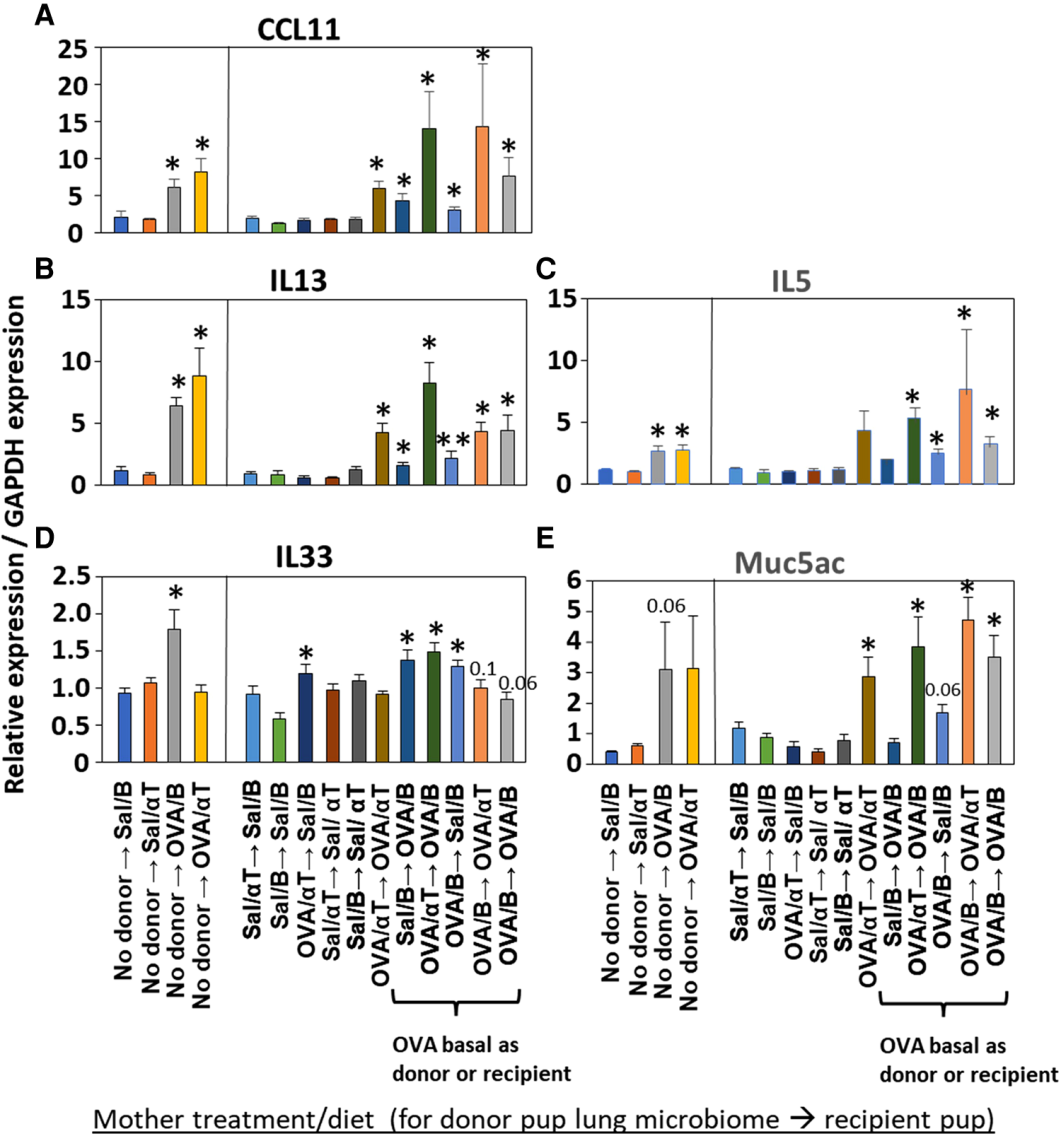
Recipient or donor microbiome from pups of allergic mothers (OVA,basal) conferred allergen-induced increases in CCL11, IL-13, IL-5, IL-33, and Muc5ac(red box). Mice were treated as in timeline in (Figure 4A). Lung cytokine expression was determined by qPCR. (**A**) CCL11. (**B**) IL-13. (**C**) IL-5. (**D**) IL-33. (**E**) Muc5ac. *N* = 6–9/group. Data are presented as mean ± SEM. *N* = 6–9/group. Sal/B, saline-treated mother with basal diet. Sal/αT, saline-treated mother with αT-supplemented diet. OVA/B, OVA allergen-treated mother with a basal diet. OVA/αT, OVA allergen-treated mother with αT-supplemented diet. *p < 0.05 as compared to the saline,basal → saline,basal group. **p < 0.05 as compared to saline/αT → saline/αT group.

Mediators of allergic inflammation were measured including serum allergen-specific antibodies, the chemokines and cytokines that mediate eosinophilia, and the mucin Muc5ac. We have reported that anti-OVA IgE is increased in the OVA/B group and this is reduced by OVA/αT (13). The serum of pups in the OVA/B → OVA/B group and OVA/B → Sal/B group had elevated anti-OVA IgE after allergen exposure (Figure 6), suggesting that the transfer of microbial communities of pups of allergic mothers with basal diet is sufficient to mediate enhanced induction of anti-OVA IgE in these pups. In contrast, there were no increases in anti-OVA IgG2b and anti-OVA IgG1 (Figure 6). The chemokine CCL11, which mediates recruitment of eosinophils, and IL-33, which is important in induction of allergic inflammation, was increased in the groups with OVA/B as donor or recipient and in the no donor → OVA/B group (Figure 7). Similarly, IL-5 and IL-13 had a significant increase in the no donor → OVA/B group and had either a trend or significant increase in most of the microbiota transfer groups with pups of OVA/B-treated moms that were either the donor or recipient of the microbe transfers (Figure 7). Muc5ac was increased in several groups that had OVA/B as donor or recipient (Figure 7). The pups with transfers of microbial communities from pups of saline-treated mothers did not have an increase in CCL11, IL-13, IL-5 or Muc5ac (Figure 7). There was also no increase in IL-33 for the recipient pups with saline-treated mothers, except a small increase for the OVA/αT → saline/B group (Figure 7). Thus, transfer of microbial communities with the OVA/B group as the donor or recipient regulated these mediators of allergic inflammation.

### A human infant plasma pro-inflammatory tocopherol isoform profile associated with altered lung microbiome

**Figure 8:**
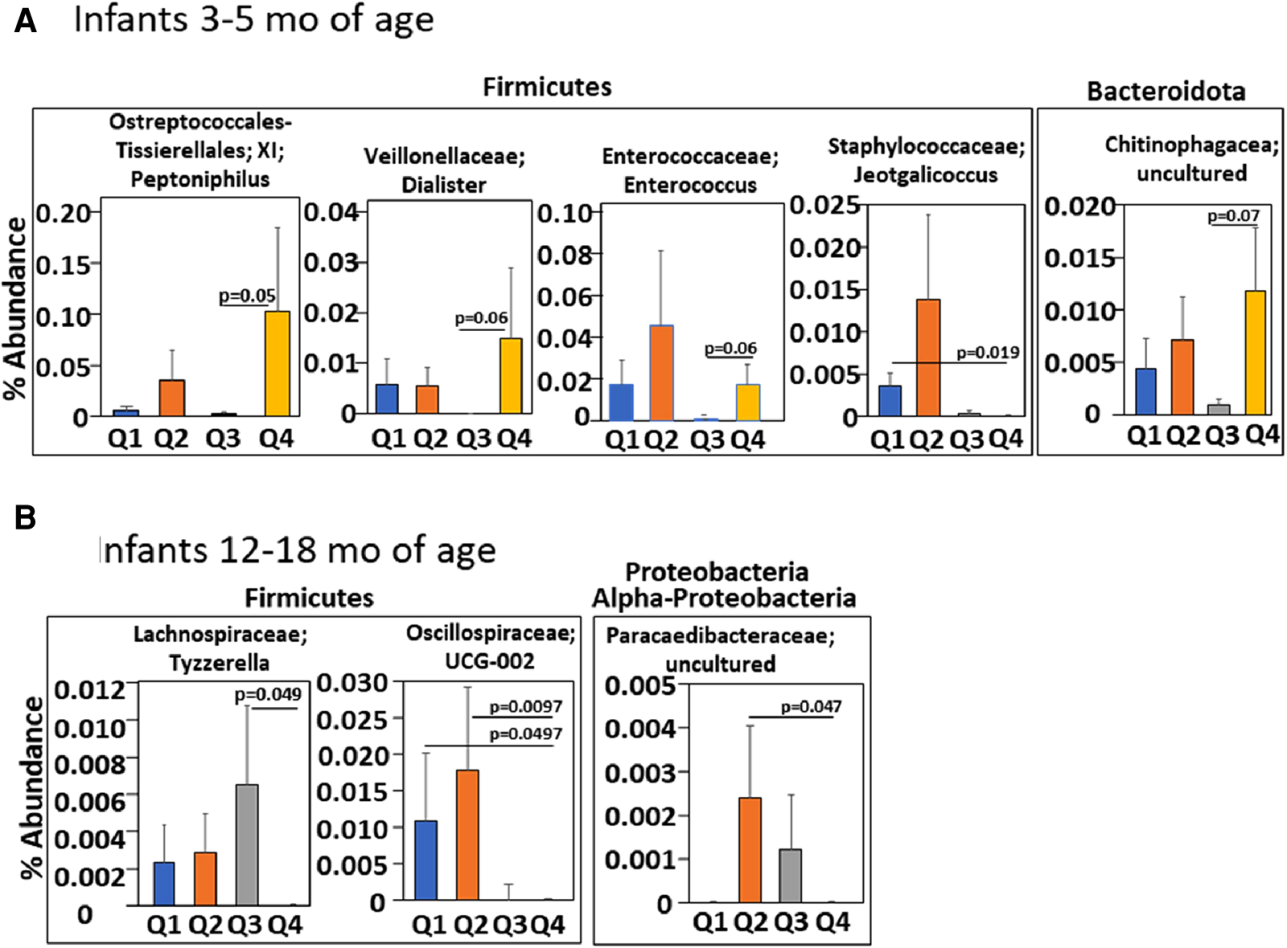
Infants with an anti-inflammatory tocopherol isoform profile (higher αT, lower γT) for allergic responses had a different abundance of bacterial microbiota compared to other tocopherol isoform profiles. Serum αT and γT for infants in the INHANCE cohort were measured by HPLC. Four groups of infants (Q1, Q2, Q3, Q4) for 3–5 months and for 12–18 months infants were generated using high and low αT and γT concentrations in (Table 1) that were defined as higher or lower than the median concentration for the tocopherol isoform for the age group. (**A**) 3–5 months and (**B**) 12–18 months of life. *p* values are given for significant differences or trends in taxa compared to group Q4.

We have demonstrated that in children and adults that better lung function associates with increasing αT concentrations when the gamma-tocopherol (γT) concentration is lower (24, 25, 65–67). To extend our microbiota studies in mice to humans, it was determined whether infants within the INHANCE cohort with an anti-inflammatory tocopherol isoform profile (high αT with low γT levels) had an altered microbiota composition compared to infants with a pro-inflammatory tocopherol isoform profile (high γT levels). To assess infant microbiome associations with an anti-inflammatory tocopherol profile, the INHANCE cohort infants that had 16S microbiota data and sufficient plasma volume for tocopherol analysis were placed in groups based on median αT and γT concentrations (64). The median serum tocopherol concentrations at 3–5 months of age were 28 µM αT and 2.6 µM γT and at 12–18 months of age were 19 µM αT and 2 µM γT (Table 1). The higher medians for 3–5 months infants are consistent with increased tocopherol concentrations during pregnancy (24) that will influence early life tocopherol concentrations in infants. The four groups are Q1 (high γT, low αT), Q2 (high γT, high αT), Q3 (low γT, low αT) and Q4 (low γT, high αT) were defined using the median serum tocopherol concentrations (Table 1). Thus, the microbiome of groups Q1, Q2 and Q3 groups were compared to Q4 because Q4 had the anti-inflammatory profile (low γT, high αT) for allergic lung inflammation, lung function and wheeze (13, 14, 24, 25, 66, 68) and had the highest lung function (64). To examine the associations of αT without elevated γT, as this was the condition in the mouse studies in (Figures 1–7), Q4 was compared to Q3. In infants 3–5 months of age, there was a significance or trend for higher % abundance in some Firmicutes and Bacteroidota taxa in Q4 compared to Q3 (Figure 8A). As infants, the airway microbiome matures from birth to 1 year of life (73–75). In INHANCE infants at 12–18 months of age, there was significantly lower % abundance in a Firmicute in Q4 compared to Q3 (Figure 8B). Moreover, for the group Q2, which has a pro-inflammatory tocopherol isoform profile with allergic lung inflammation and function (13, 14, 24, 25, 66, 68), there was a significantly higher % abundance in taxa of a Firmicute and an Proteobacteria (Figure 8B). These data suggest that tocopherol profiles associate with altered microbiome abundance of several taxa in infants.

**Table 1:** Grouping of infants by median serum αT and γT concentrations in the INHANCE cohort. Serum αT and γT for infants in the INHANCE cohort were measured by HPLC. Median serum αT and γT concentrations for infants at (**A**) 3–5 months and (**B**) 12–18 months of life. Four groups Q1, Q2, Q3, and Q4 of infants for 3–5 months and for 12–18 months infants were generated using high and low αT and γT concentrations defined as higher or lower than the median for the tocopherol isoform for the age group. *N*, number of participants in group.

Group	αT (µM)	γT (µM)	*N*
(**A) 3–5 months, serum tocopherol**
Q1	<28	>2.6	7
Q2	>28	>2.6	15
Q3	<28	<2.6	13
Q4	>28	<2.6	8
**(B) 12–18 months, serum tocopherol**
Q1	<19	>2	11
Q2	>19	>2	14
Q3	<19	<2	11
Q4	>19	<2	14

## Discussion

We report that pups of allergic mothers had allergic lung inflammation and lung microbial community dysbiosis with increased Proteobacteria and decreased Bacteroidota before and after allergen sensitization. This indicated a sustained dysbiosis in the pups of allergic mothers. The generation of lung microbe community dysbiosis was blocked by supplementation of the mothers with αT during gestation and nursing. We also demonstrated a functional effect of the microbiome dysbiosis. Fascinatingly, in studies with BAL microbe community transfers, the lung microbiome dysbiosis of neonates of allergic mothers mediated enhanced neonate responsiveness to allergen with increases in eosinophils, monocytes, lymphocytes, CCL11, IL-5, IL-13, IL-33 in the lungs of recipient pups. There was also increased serum anti-OVA IgE. In contrast, neonates of allergic mothers were not protected from development of allergy by the transfer of non-dysbiotic microbial communities from either neonates of non-allergic mothers or neonates of αT-supplemented allergic mothers. These data suggest that the dysbiotic lung microbiome is dominant and sufficient for enhanced neonate responsiveness to allergen. Furthermore, human infants with an anti-inflammatory tocopherol profile compared to a pro-inflammatory tocopherol profile had an altered abundance of several Proteobacteria, Firmicutes and Bacteriodota taxa.

In humans, the onset of atopy and asthma correlates with home environment, viral infections, and antibiotic exposures (76). It has been suggested that the diversity of overall bacterial environmental exposures rather than any one exposure may be contribute to immune skewing and allergen responses (77). Human adults with allergic asthma have airway microbe community dysbiosis with decreased Bacteroides and increased Firmicutes and Proteobacteria (78–81). This includes an increase in the Proteobacteria *Haemophilus* in asthmatics (82). The increase in abundance of Proteobacteria, including *Haemophilus* and *Moraxella,* is also observed in adult patients with neutrophilic asthma and the abundance of these microbiota was associated with asthma severity (83). In another study of induced sputum from patients with severe asthma, an enrichment of *Moraxella, Haemophilus*, or Streptococcea associated with severe airway obstruction and airway neutrophilia (84). Because multiple bacterial taxa associate with regulation of allergic or neutrophilic asthma, it suggests that individual taxa of the bacterial microbial community in the lung may be less important than the interactions of bacteria in general or shared features of the bacteria. A similar airway microbial community is present in infants with wheeze. In mice and human infants, the nasal and lung airway differ in bacteria species, but are similar at the family level (85), suggesting that there may be a similar regulation or function of the species in allergic airway inflammation. It has been reported that PND6 neonatal mice have a predominance of Firmicutes and gamma-Proteobacteria in the lung but then as adults, mice acquire an increase in abundance of Bacteroidota (72). In IL13-transgenic adult mice with allergic lung inflammation, there is increased Proteobacteria and decreased Bacteroides in airways (69). Similarly, in infants, hypopharyngeal *Streptococcus pneumoniae*, *Moraxella catarrhalis*, and/or *Haemophilus influen*zae at 1 month of age associated with persistent wheeze, increased blood eosinophil counts and elevated total IgE at age 4, and asthma diagnosis at age 5 (33). In children, infection of *M. catarrhalis* or *S. pneumoniae* and rhinovirus associated with greater severity of respiratory illness, including asthma exacerbations, suggesting that respiratory bacteria may contribute to airway inflammation (86). In our studies, we demonstrated that the neonatal mice of allergic mothers have an altered BAL microbiome with increased Proteobacteria and decreased Bacteroidota as early as postnatal day 4. Then after allergen challenge, there was also a microbiome dysbiosis with a combination of increased Proteobacteria or Firmicutes and decreased Bacteroidota; this is consistent with the reports of associations of increased Proteobacteria and decreased Bacteroides in infants with wheeze (34). We also demonstrated that Shannon alpha-diversity was reduced in groups with allergic inflammation and that the groups with allergic inflammation separated in the PCA plot of the Bray-Curtis beta-diversity. These data are consistent with studies demonstrating changes in airway microbiota community diversity in subjects with wheeze or asthma (62, 87).

Reports have demonstrated airway dysbiosis with allergic asthma but have not assessed whether lung microbial community dysbiosis has a functional effect on allergen sensitization. To go beyond associations of lung microbe composition with allergic lung inflammation in neonates, analyses of the function of lung microbiome in neonates are necessary. The function of microbial communities in tissues has mostly been studied in the gut, including mouse models with the transfer of gut microbial communities in disease states or in germ-free conditions. In an adult mouse model, administration of *Escherichia coli* to the lung can skew the type of Th responses and protect the mice from induction of allergic airway inflammation (88). Also, Herbst and colleagues reported that induction of allergy in germ-free neonatal mice was protected by colonization by co-housing with specific-pathogen-free (SPF) non-allergic mice before allergen sensitization, suggesting a potential protective effect of microbiota of SPF mice, although this was colonization with microbiota composition of non-allergic mice (89). However, the function of this control microbiota in non-germ-free conditions is not known. In non-germ-free conditions, microbe transfers have been studied in adult mice that were most often pretreated with antibiotics to disrupt the microbe taxa abundance and provide a niche for transferred microbes. In our studies with SPF conditions, antibiotics were not needed as the PND4 pups are rapidly growing neonates, likely providing niches for establishment of the transferred microbial communities. In our studies, transfer of the dysbiotic microbial communities from pups of allergic mothers to pups of non-allergic mothers established a dysbiotic microbial composition in the recipient pups and increased responsiveness to allergen. Thus, the dysbiotic microbial communities of neonates is sufficient for enhanced responsiveness to allergen and a dysbiotic microbial composition is sustained in recipient pups through the allergen challenge. However, the transfer of microbiome from pups not susceptible to allergen hyperresponsiveness (neonates of non-allergic mothers) to pups of allergic mothers did not protect the pups of allergic mothers from hyperresponsiveness to allergen.

Early life development of asthma and allergic disease results from complex interactions of genetic and environmental factors (90), including the dietary lipids, tocopherols (13, 14). In adults and children, increasing plasma αT concentrations associates with better lung function and γT associates with lower lung function (24, 25, 65, 67, 68). In mechanistic studies in adult and neonate mouse models of allergic lung inflammation, αT blocks development of lung eosinophilia and this is counteracted by γT (13–15, 21, 46, 68). In mechanistic studies, αT blocks the development of allergic inflammation in adults and neonates, at least, through functioning as an antagonist for protein kinase C during VCAM-1 signaling in endothelial cells, thereby, blocking VCAM-1-dependent eosinophil recruitment into tissues (15, 22, 91). αT also blocks development of subsets of dendritic cells involved in allergic disease (CD11c ^+ ^CD11b^+^ DCs) *in vitro* and *in vivo* (13, 18). In our studies herein, maternal supplementation with αT blocked development of microbiome dysbiosis and allergen-induced lung eosinophilia in offspring of allergic mothers. However, transfer of microbial communities from pups of non-allergic mothers or from allergic mothers with αT-supplemented diet did not block microbial community dysbiosis or the hyperresponsiveness to allergen, as detected by increased BAL eosinophilia when the recipient pups were from allergic mothers with a basal diet. This suggests that the microbiome of offspring of allergic mothers is dominant. Consistent with a dominant microbiome profile, the transfer of BAL microbial communities from pups of allergic mothers to recipient pups of allergic mothers or non-allergic mothers with or without αT yielded a dysbiotic lung microbial composition and responses to allergen challenge with development of BAL eosinophilia.

Inflammation with eosinophilia is regulated by the chemokines CCL11 and the cytokines IL-13, IL-5, and IL-33. These mediators were induced in the no donor → OVA/B group of pups; in these studies only IL-33 expression was blocked by maternal supplementation of allergic mothers with αT (no donor → OVA/αT group). Because CCL11, IL-13 and Muc5ac are produced by airway epithelium, it suggests that αT did not intervene in epithelial-generated mediators of allergic inflammation. In pups with lung microbiome transfers, all pups with either microbiome of OVA/B group as donor or recipients had increases in several of the mediators of allergic inflammation, including CCL11, IL-13, IL-5 or IL-33. This indicates that in the presence of the microbial community dysbiosis, there was elevated responsiveness to allergen by increasing multiple allergen-induced mediators of allergic inflammation. Some variation in effects on CCL11, IL-13, IL-5 and IL-33 in the microbiome transfer studies with donor or recipient pups of the OVA/αT group may result from αT effects on cell signaling because besides αT anti-oxidant functions, αT is also an antagonist of PKCs by binding the regulatory domain of PKCs (15, 18, 20–23). This suggests that maternal αT has some effects on cytokine expression that may, in part, be independent of the transferred dysbiotic microbiota. We also found that recipient pups of allergic mothers on basal diet (OVA/B) had increased serum anti-OVA IgE, except for the pups of the OVA/αT → OVA/B group. However, suggesting although there were low undetectable levels of serum anti-OVA IgE, anti-OVA specific IgE bound to FcεR on leukocytes in tissues may participate in the allergic response to allergen in this group of pups. It is suggested that in humans, serum IgE does not always associate with severity of allergic asthma and allergic diseases (92, 93). Allergen-specific IgE-mediated responses can occur at low serum levels of allergen-specific IgE when there is allergen-specific IgE bound to FcεR on leukocytes in the tissue (94, 95) Thus, supplementation of allergic mothers with αT not only blocked the development of allergic inflammation in mouse neonates, but also blocked the development of neonate lung dysbiosis.

 In the airway microbial composition of infants in the INHANCE cohort (62, 63), nasal microbiome diversity at 3 months of age is lower in the infants with wheeze in the first year of life, with a decrease in *Corynebacterium* and increase in the proteobacteria *Moraxella* (62). Increased *Moraxella* is seen in episodes of wheeze (35, 96–100)*.* Also, lung function in children associates with profiles of tocopherol isoforms (24). But it was not reported if the tocopherol isoform profiles early in life associate with infant airway microbiome composition. In our analyses of the INHANCE cohort, infants at 3–5 months of age in group Q4 with an anti-inflammatory tocopherol profile of higher αT and lower γT (13–15, 21, 24, 25, 46, 65, 67, 68) had a significant lower abundance of the family Staphylococcaceae genus *Jeotgalicoccus* as compared to the opposing group Q1 that had a proinflammatory profile of tocopherol isoforms with low αT high γT (13–15, 21, 24, 25, 46, 65, 67, 68). Because Staphylococcaceae in the lung has been associated with asthma (32), group Q1 might associate with development of asthma later in life, but this will need further longitudinal study. Additionally, group Q4 had a significantly higher % abundance of the phyla Firmicute genus *Enterococcus* as compared to group Q3 which has a low αT low γT. The *Enterococcus* genus are present as commensals in lung and gut and Enterococcus can limit the growth of pathogenic bacteria, although outgrowth of some *Enteroccocus spp.* can be pathogens (101–104). The 3–5 months old infants in group Q4 also had a significantly higher % abundance for the phyla Firmicute genus *Peptoniphilus* and a trend for higher % abundance of the genus *Dialister* as well as the Bacteriodota family Chitinophagaceae as compared to group Q3. *Peptoniphilus* genus is present in a healthy microbiome community of the nasopharynx (105) and is decreased in the nasopharynx with disease such as chronic rhinosinusitis (106). *Diallister spp.* are enriched in healthy human lung compared to lung with infection (107) and is increased in nasopharynx for adult subjects with non-exacerbated asthma as compared to exacerbated asthma (106, 108). Chitinophagaceae are present in myconium and the lung (109–111). Moreover, Chitinophagaceae are considered beneficial bacteria in the lower respiratory tract because Chitinophagaceae limit colonization by pathogens in animal models (111–113). Thus, the higher abundance in these taxa in group Q4, as compared to group Q3 at 3–5 months of age, is consistent with an anti-inflammatory tocopherol isoform profile and is consistent with a potential for lower airway disease.

In infants at age 12–18 months in the INHANCE cohort, there was a significantly lower % abundance in a Firmicute family Lachnospiraceae in group Q4 compared to group Q3. This was similar to our neonate mouse studies with αT supplementation demonstrating less Lachnospiraceae and less allergic lung inflammation. In the 12–18 months age group, group Q4 had a lower abundance of phyla Firmicutes family Oscillospiraceae and phyla Proteobactera class Alpha-proteobacteria family Paracaedibacteraceae as compared to group Q2, that has a pro-inflammatory tocopherol isoform profile for allergic inflammation. Interestingly, Oscillospiraceae are not detected in lung until inflammation is induced by TLR stimulation in mice and rats (114, 115). This is interesting because TLR stimulation plays a role in allergic responses (116). Proteobacteria are elevated in infant lungs with increased prevalence of wheeze (62) and in our neonatal mice with increased allergic lung inflammation. These differences in group Q4 and group Q2 bacterial microbiota suggest that group Q2 has a microbial community that may have an increased potential for elevated airway inflammation as compared to group Q4. A limitation of the INHANCE study is use of the readily accessible infant upper airway microbiome as a surrogate of microbiome in lower airways as these sites have both microbiome similarities and differences, as previously discussed (62, 85). Nevertheless, the studies of upper airway in infants and adults and the studies of lower airway in children and adults suggest that there is a lower microbiota diversity and increased Proteobacteria with wheeze or asthma (62, 78–81).

In conclusion, unbiased analyses of the lung microbial communities of mouse neonates of allergic mothers indicate that both before and after allergen challenge, there is an altered lung bacterial microbiome composition that is consistent with that found in infants with wheeze and adults with allergic asthma. Most importantly, we have gone beyond associations of lung microbial composition dysbiosis with allergic lung inflammation and have demonstrated that the lung microbiome composition of offspring of allergic mothers confers neonate responsiveness to allergen and development of allergic disease in mouse models. Thus, an early life airway microbiota dysbiosis may have a significant function in development of wheeze and allergic asthma in children. This is a novel regulatory mechanism for development of responses to allergen challenge early in life that may inform design of future studies for approaches in the prevention or intervention in asthma and allergic disease. Further mechanisms for microbiome regulation are currently under investigation by our research group. Moreover, the development of lung microbial community dysbiosis in the neonatal mice was blocked by maternal dietary supplementation with αT during pregnancy and nursing, suggesting a potential target for intervention early in life. In human infants at 3–5 months and at 12–18 months of age, there was an anti-inflammatory tocopherol isoform profile associated with microbiome taxa abundance composition that may have the potential to limit development of airway disease.

## Data Availability

The raw fastq files of the 16S rRNA analysis from mouse BAL in Figures 2–5 are deposited as NCBI BioProject repository, accession number ID PRJNA925891. The raw fastq files of the 16S rRNA analysis in INHANCE cohort in Figure 8 are deposited as NCBI BioProject repository, accession number ID PRJNA928382.

## Supplementary material

SUPPLEMENT FIGURE 1

Alpha-diversity of donor pup BAL microbiota and allergen-challenged recipient pup microbiota. Data are from experiments in Figures 2, 3 and 4. Shannon Index for alpha-diversity which incorporates the total number of bacterial species and relative differences in the abundance of taxa in the community. (**A**) PND4 donor BAL microbiota communities. (**B**) PND16 BAL microbiota communities of allergen-challenged pups. Sal/B, saline-treated mother with basal diet. Sal/αT, saline-treated mother with αT-supplemented diet. OVA/B, OVA allergen-treated mother with a basal diet. OVA/αT, OVA allergen-treated mother with αT-supplemented diet. *. *p* < 0.045 for decrease as compared to no donor → Sal/B group. **. *p* < 0.035 for decrease as compared to Sal/B → Sal/B group. ^+^. *p* < 0.04 for decrease as compared to no donor → Sal/αT group. ^++^. *p* < 0.045 for decrease as compared to OVA/αT → OVA/αT group.

SUPPLEMENT FIGURE 2

Beta-diversity of donor pup BAL microbiota and allergen-challenged recipient pup microbiota. Data are from experiments in Figures 2, 3 and 4. PND4 Donor pup and PND16 recipient pup BAL 16S microbiota were analyzed for beta-diversity with donor and transfer microbiome groups. The donor and transfer groups are displayed in separate three-dimension graphs for clarity but were generated by the same Principal Component Analyses (PCA). (**A**) PCA plots of the Bray-Curtis analysis that generated a dissimilarity distance matrix for microbiota between groups. Bray-Curtis dissimilarity between groups is a measure that incorporates both overall abundance per sample and abundance of each taxa of the microbiota communities. (**B**) PCA plots of the Weighted-Unifrac analyses between groups which incorporates the relative abundance of taxa shared between samples. (**C**) PCA plots of Unweighted-Unifrac analyses between groups which only incorporates the presence/absence of taxa. Sal/B, saline-treated mother with basal diet. Sal/αT, saline-treated mother with αT-supplemented diet. OVA/B, OVA allergen-treated mother with a basal diet. OVA/αT, OVA allergen-treated mother with αT-supplemented diet.
