# Supplementary figures and images for: Dysbiotic lung microbial communities of neonates from allergic mothers confer neonate responsiveness to suboptimal allergen
Source: Front Allergy. 2023 Mar 10;4:1135412. doi: 10.3389/falgy.2023.1135412 (PMC10036811; doi:10.3389/falgy.2023.1135412)

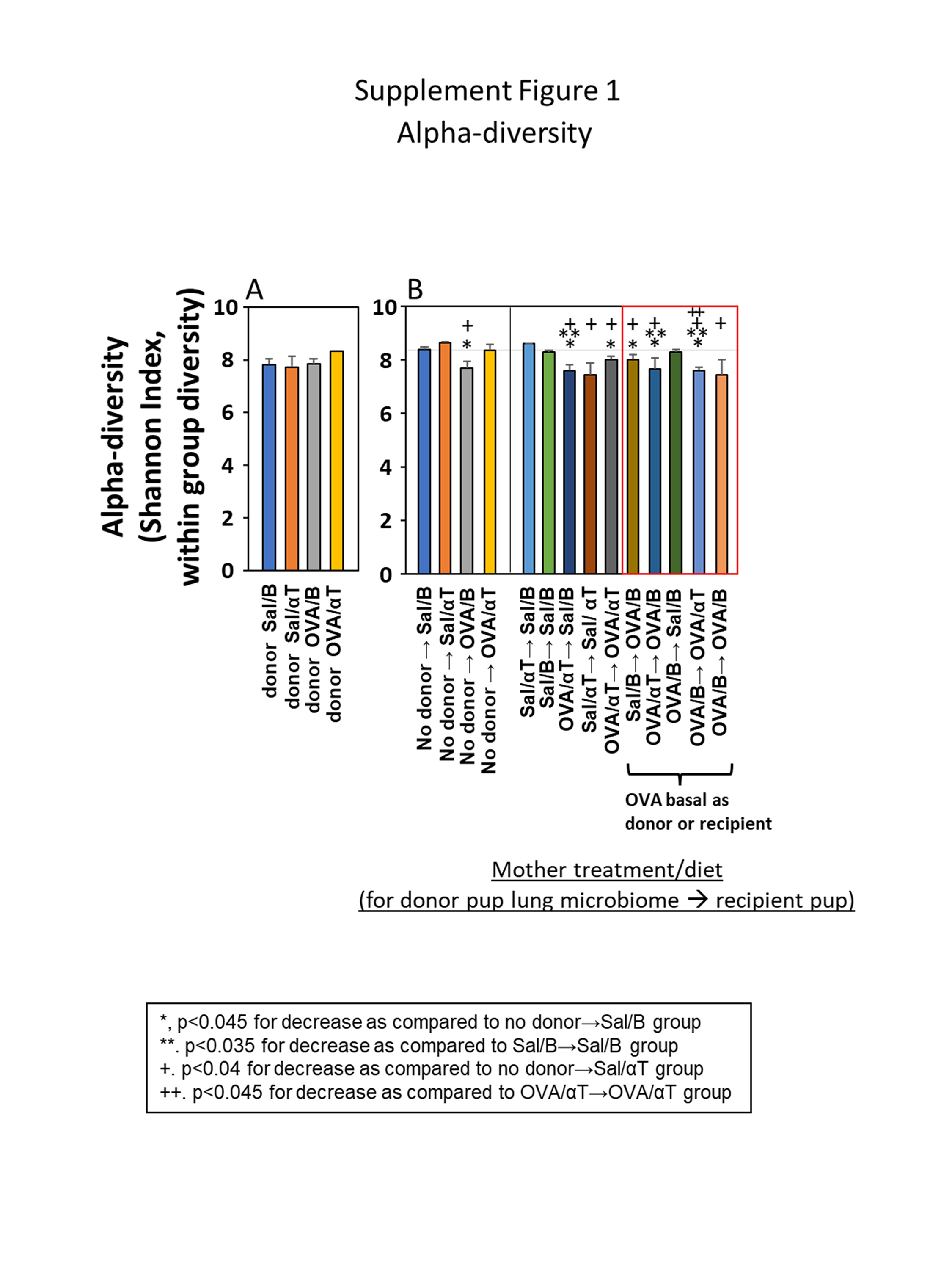

Supplement: Supplementary file 1 [file Image1.tif]

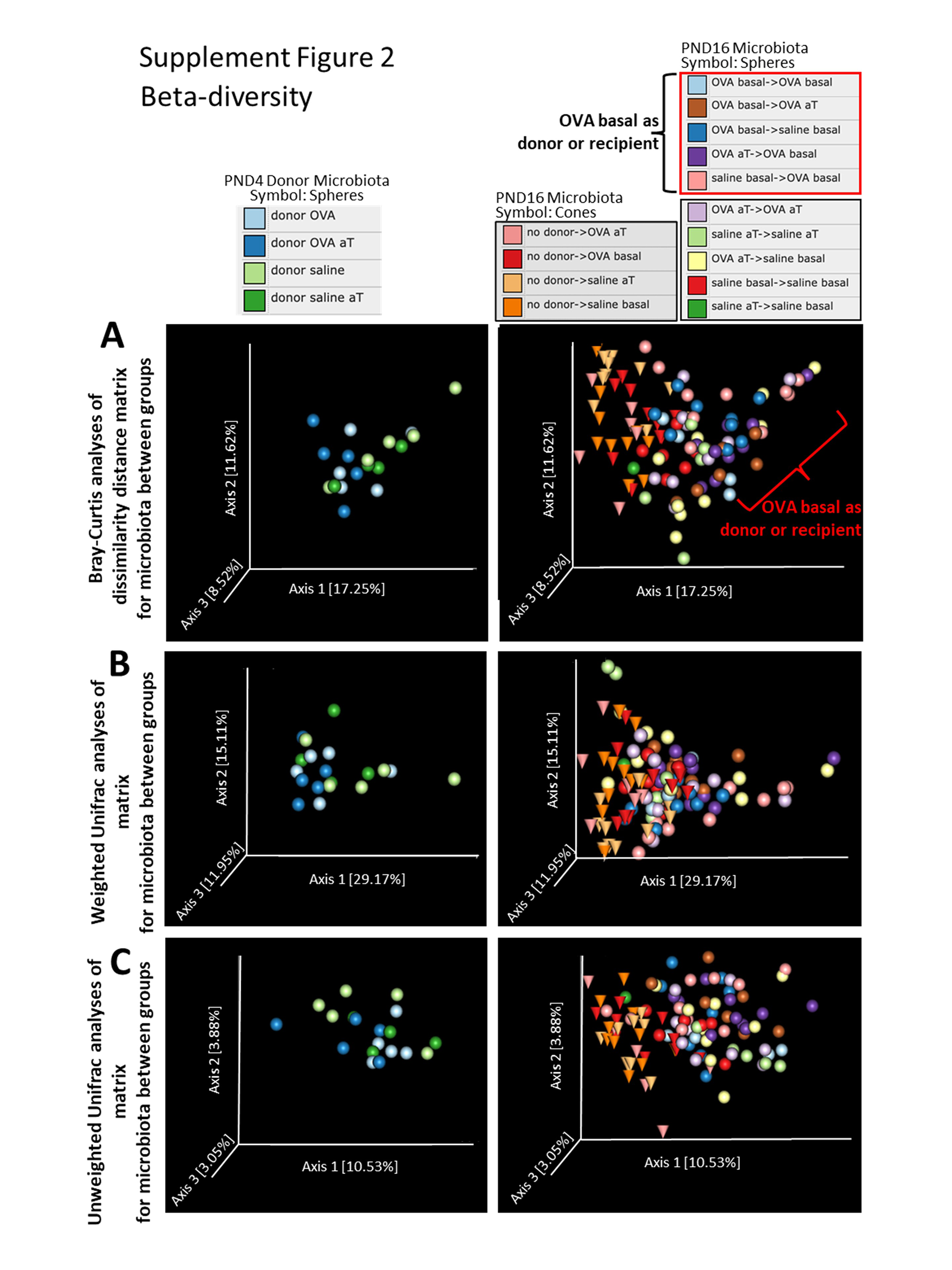

Supplement: Supplementary file 2 [file Image2.tif]
